# From Flower to Medicine: Green-Synthesized Silver Nanoparticles as Promising Antibacterial Agents

**DOI:** 10.3390/ph18050691

**Published:** 2025-05-07

**Authors:** Mohd Saeed, Reem Binsuwaidan, Nawaf Alshammari, Ahmed M. Alharbi, Nadiyah M. Alabdallahd, Nawaf A. Alotaibi, Samra Siddiqui, Safia Obaidur

**Affiliations:** 1 Department of Biology, College of Science, University of Hail, Hail 55476, Saudi Arabia; naib.alshammari@uoh.edu.sa; 2 Department of Pharmaceutical Sciences, College of Pharmacy, Princess Nourah Bint Abdulrahman University, Riyadh 11671, Saudi Arabia; rabinsuwaidan@pnu.edu.sa; 3 Department of Medical Laboratory Science, College of Applied Medical Sciences, University of Hail, Hail 81422, Saudi Arabia; am.alharbi@uoh.edu.sa; 4 Department of Biology, College of Science, Imam Abdulrahman Bin Faisal University, P.O. Box 1982, Dammam 31441, Saudi Arabia; nmalabdallah@iau.edu.sa (N.M.A.); 2240500250@iau.edu.sa (N.A.A.); 5 Basic & Applied Scientific Research Centre, Imam Abdulrahman Bin Faisal University, P.O. Box 1982, Dammam 31441, Saudi Arabia; 6 Department Health Services Management, College of Public Health and Health Informatics, University of Hail, Hail 81422, Saudi Arabia; s.siddiqui@uoh.edu.sa; 7 Department of Clinical Laboratory Sciences, College of Applied Medical Sciences, King Khalid University, Abha 61421, Saudi Arabia

**Keywords:** hibiscus rosa sinensis (HRS), silver nanoparticles (AgNPs), anti-bacterial, anti-biofilm, anti-cancer, reactive oxygen species

## Abstract

**Background:** Breast cancer and chronic bacterial infections are pressing global health issues, and traditional treatments are often hampered by resistance and adverse side effects. This study sought to create silver nanoparticles (AgNPs) through eco-friendly synthesis using Hibiscus rosa sinensis (HRS) flower extract and to assess their antibacterial, antibiofilm, and anticancer properties. **Methods:** HRS extract functioned as both a reducing and stabilizing agent in the synthesis of AgNPs. The nanoparticles were characterized using ultraviolet–visible spectroscopy (UV–Vis), Fourier-transform infrared (FTIR) spectroscopy, and transmission electron microscopy (TEM). Antibacterial and antibiofilm properties were evaluated against gram-positive (*Staphylococcus aureus* and *Enterococcus faecalis*) and gram-negative (*Escherichia coli* and *Pseudomonas aeruginosa*) bacteria using agar well diffusion and XTT reduction assays. The cytotoxic effects on MDMB-231 breast cancer cells and normal splenocytes were measured using the MTT assay, whereas fluorescence microscopy was used to observe reactive oxygen species (ROS) production, changes in mitochondrial membrane potential, and caspase-3 activation. **Results:** The synthesized HRS-AgNPs, primarily ranging from 10 to 50 nm, displayed a distinct surface plasmon resonance (SPR) peak at 428 nm. They exhibit notable antibacterial activity, especially against gram-positive bacteria, and effectively disrupt bacterial biofilms. Cytotoxicity evaluations showed that HRS-AgNPs decreased the viability of MDMB-231 cells in a dose-dependent manner, with minimal toxicity observed in normal splenocytes. The increase in ROS levels, reduction in mitochondrial membrane potential, and heightened caspase-3 activity collectively suggest apoptosis-driven cell death in cancer cells. **Conclusions:** HRS-AgNPs demonstrated dual functionality, with strong antibacterial and selective anticancer effects. Their environmentally friendly synthesis, stability, and significant biological activities suggest their potential for further development, including in vivo safety and efficacy assessments for clinical applications in treating infections and breast cancer.

## 1. Introduction

Infection and cancer are two leading threats to human health and lifestyles. Both present significant challenges to healthcare systems worldwide, demanding innovative and multifaceted therapeutic strategies.

The establishment and proliferation of microbial biofilms on biotic and abiotic surfaces represent a formidable challenge in both clinical and industrial settings. Biofilms are complex communities of microorganisms encased in a self-produced extracellular polymeric matrix (EPS) and are notorious for their recalcitrance to conventional antimicrobial therapies and their implication in a wide array of persistent and chronic infections [1,2]. The initial colonization of surfaces by planktonic bacteria, followed by the secretion of EPS, results in a robust architecture that physically impedes the diffusion of antimicrobial agents and confers enhanced resistance to host immune responses [3,4]. This phenomenon has been observed in a wide variety of Gram-positive and Gram-negative bacterial species, including *Enterococcus faecalis*, *Staphylococcus aureus*, *Streptococcus viridans*, *Escherichia coli*, *Klebsiella pneumoniae*, *Proteus mirabilis*, *and Pseudomonas aeruginosa* [2,5,6,7]. Notably, *S. aureus* and *S. epidermidis* are of particular concern, as they are responsible for a significant proportion of device-related infections, accounting for 40–50% of prosthetic heart valve infections, 50–70% of catheter-associated infections, and an alarming 87% of bacteremia cases [8]. The clinical impact of biofilm formation extends beyond the confines of medical devices such as catheters, implants, heart valves, stents, suture materials, and contact lenses. Biofilms are ubiquitous in diverse environments, including the human and animal gastrointestinal tract, wastewater treatment systems, marine pipelines, ship hulls, and the oil industry. Within the human host, biofilms are implicated in an estimated 80% of microbial infections, contributing to the pathogenesis of a spectrum of diseases, including endocarditis, cystic fibrosis, periodontitis, chronic wounds, meningitis, kidney infections, and infections associated with prosthetic and implanted devices [9,10].

Simultaneously, the worldwide prevalence of breast cancer has become a concern and presents a substantial challenge to public health. In 2023, breast cancer emerged as the most diagnosed cancer worldwide, overtaking lung cancer, with an estimated 2.3 million new cases reported each year, accounting for 12% of all cancer diagnoses [11,12].

The distinct etiologies of biofilm-related infections and breast cancer highlight the need for innovative multifaceted therapeutic strategies to address these intricate and widespread healthcare issues. These two issues, which may initially appear unrelated, exemplify the persistent challenge of managing complex health threats effectively. The need to address biofilm formation has been increasingly underscored by the growing global challenge of antimicrobial resistance (AMR). Furthermore, recent studies have clarified the detrimental impact of pathogenic biofilm formation, especially on indwelling medical devices like ventilators in COVID-19 patients, in undermining immune function and leading to considerable morbidity and mortality [13,14]. Similarly, chemotherapeutic agents, including cyclophosphamide, cisplatin, doxorubicin, and docetaxel, constitute the fundamental components of breast cancer therapy; their effectiveness is frequently compromised by significant adverse effects and the development of drug resistance [15,16].

The need for innovative therapeutic strategies to address both biofilm-related infections and cancer has led to the exploration of nanotechnology as a promising avenue. Nanomaterials, with their unique physicochemical properties, offer distinct advantages in overcoming the limitations of conventional therapies. Their nanoscale dimensions, high surface-area-to-volume ratio, and enhanced reactivity have demonstrated remarkable efficacy against multidrug-resistant infections and biofilms, as well as in oncology, where nanotechnology-based therapies have revolutionized cancer treatment by enabling precision-targeted interventions that minimize systemic toxicity [17,18,19]. Nanomaterials have demonstrated remarkable efficacy against multidrug-resistant infections and biofilms. In oncology, nanotechnology-based therapies have revolutionized cancer treatment by enabling precision-targeted interventions that minimize systemic toxicity. This dual applicability underscores the immense potential of nanotechnology in addressing critical challenges in both biofilm management and cancer therapy.

The increasing prevalence of environmental issues has led to a significant interest in the formulation of environmentally sustainable approaches for the synthesis of nanomaterials. Green synthesis, particularly using plant-mediated methodologies, has emerged as a noteworthy alternative to traditional synthetic pathways. The synthesis of nanomaterials via plant-based methods presents several advantages, such as compatibility with environmental standards, biocompatibility, cost-effectiveness, and improved safety profiles, which are realized through the exclusion of hazardous chemicals. The use of various plant constituents, including leaves, fruits, stems, and roots, facilitates the synthesis of nanoparticles with specific sizes, morphologies, and compositions. The integration of nanotechnology and phytomedicine presents a significant opportunity for the development of sophisticated therapeutic agents with synergistic properties. Silver nanoparticles (AgNPs) represent a class of nanomaterials that have garnered considerable attention owing to their unique physicochemical properties that are intrinsically linked to the size, morphology, and dielectric characteristics of the surrounding microenvironment. A defining characteristic of AgNPs is their ability to demonstrate localized surface plasmon resonance (LSPR), a phenomenon that results from the coherent oscillation of conduction-band electrons upon interaction with incident electromagnetic radiation. The localized surface plasmon resonance (LSPR) effect endows silver nanoparticles (AgNPs) with considerable versatility, thereby enabling their utilization across a wide range of domains, including targeted drug delivery, nanomedicine, electronics, photonics, optical engineering, catalysis, antimicrobial formulations, environmental remediation, therapeutic interventions, and advanced sensor technologies [11,20,21,22]. The synthesis of silver nanoparticles (AgNPs) has been thoroughly documented using a range of chemical, physical, and biological methodologies. Among these methods, biogenic synthesis has emerged as a promising and environmentally sustainable strategy. The methodologies employed utilize the inherent reducing and stabilizing properties of biomolecules derived from plant extracts, proteins, carbohydrates, and microbial secondary metabolites. The biomolecules exhibit a high density of functional moieties, including carbonyl (C=O), carboxyl (COOH), and hydroxyl (–OH) groups. The functional groups serve as electron donors, facilitating the reduction of silver ions (Ag^+^) to zerovalent silver (Ag^0^) and are integral to the stabilization of nascent nanoparticles [23,24,25,26].

*Hibiscus rosa-sinensis* (HRS), a significant species within the Malvaceae family commonly referred to as the Chinese rose, is widely acknowledged for its diverse array of medicinal attributes. Phytochemical analysis of *H. rosa-sinensis* has revealed a diverse array of bioactive compounds such as flavonoids, tannins, terpenoids, saponins, and alkaloids, which are hypothesized to play a significant role as therapeutics. Recent studies have supported the historical applications of *H. rosa-sinensis* as extracts derived from different parts of the plant exhibit a wide range of pharmacological effects. These effects include hypotensive, antipyretic, anti-inflammatory, antineoplastic, antioxidant, antibacterial, antidiabetic, wound-healing, and abortifacient properties. In addition to its therapeutic applications, *H. rosa-sinensis* is frequently incorporated into dietary practices, as a herbal infusion commonly known as hibiscus tea. Furthermore, hibiscus tea and its extracts have been extensively employed in ethnomedical practices for the treatment of various conditions, including hypertension, dyslipidemia, and cancer progression, highlighting their potential as versatile and multifaceted natural remedies [27,28,29]. Beyond its traditional medicinal uses, *Hibiscus rosa sinensis* has garnered increasing attention for its anticancer properties. Numerous studies have demonstrated that extracts or specific phytoconstituents derived from *H. rosa sinensis* can effectively inhibit the viability and proliferation of human breast cancer cells, including MCF-7 and triple-negative breast cancer lines [30,31,32,33]. These anticancer effects are primarily attributed to mechanisms such as the induction of apoptosis, disruption of mitochondrial membrane potential, and cell cycle arrest in various in vitro models [30,31]. These promising findings underscore the translational potential of *H. rosa sinensis*-derived agents in breast cancer therapy. Furthermore, they validate our approach of utilizing *H. rosa sinensis* extract in the green synthesis of silver nanoparticles for anticancer investigations. By leveraging the bioactive properties of *H. rosa sinensis*, we aim to develop novel nanotherapeutic strategies that harness its natural anticancer potential for enhanced therapeutic outcomes. This research aims to assess the antibacterial, antibiofilm, and cytotoxic capabilities of these biosynthesized nanoparticles, with a particular emphasis on their effectiveness against breast cancer. This investigation contributes to the advancement of innovative therapeutic approaches for combating biofilm-associated infections and breast cancer, addressing two critical and widespread healthcare issues.

## 2. Results and Discussion

### 2.1. HRS Flower Extract-Mediated Synthesis of AgNPs

This study explores the viability of utilizing Hibiscus flower extract as both a reducing as well as a stabilizing agent in the environmentally friendly synthesis of AgNPs. Upon introducing the aqueous Hibiscus flower extract to a solution containing Ag^2+^, a notable chromatic shift was observed, with the mixture progressing from a deep pink to a rich brown coloration. This visual transformation is indicative of the Surface Plasmon Resonance (SPR) phenomenon, a distinctive optical characteristic associated with colloidal AgNPs. The SPR effect results from the synchronized oscillation of conduction-band electrons when exposed to electromagnetic radiation, serving as an initial yet conclusive sign of successful nanoparticle generation [34,35].

### 2.2. UV–Vis-NIR Spectra of AgNPs

**Figure 1:**
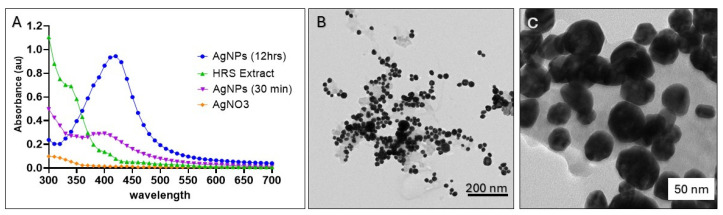
Characterization of AgNPs synthesized using *Hibiscus rosa sinensis* (HRS) extract. (**A**) UV–Vis spectra of AgNPs synthesized at different time intervals (12 h and 30 min) and with silver nitrate (AgNO_3_) as a control. (**B**) Transmission electron microscopy (TEM) image of AgNPs illustrating their morphology and size distribution. (**C**) High-resolution TEM image of AgNPs demonstrating their crystalline structure.

Characterization of biogenic HRS-AgNPs through UV–visible absorption spectroscopy serves as a critical analytical methodology for the assessment of the formation, stability, and optical characteristics of metal nanoparticles (NPs) in aqueous environments. The absorption spectrum of metallic nanoparticles, specifically the position and intensity of the Surface Plasmon Resonance (SPR) band, exhibits significant sensitivity to various parameters, including particle size, morphology, dielectric environment, and interparticle interactions, notably agglomeration [24,36]. Consequently, the bioreduction of silver ions (Ag^+^) can be precisely monitored in situ using UV–VIS spectrophotometry. The silver nanoparticles (AgNPs) synthesized in this study demonstrated a distinct absorption peak at approximately 428 nm, as illustrated in Figure 1A. This observation aligns with prior studies, which have established that small (10–50 nm), spherical, or near-spherical silver nanoparticles typically exhibit surface plasmon resonance (SPR) peaks within the 400–450 nm range [22,37]. For instance, Morones et al. [22] reported a prominent SPR peak near 420 nm for silver nanoparticles of comparable size, while Carlson et al. [37] noted that minor variations in peak position can arise due to factors such as particle shape, size distribution, and the nature of capping agents. The 428 nm SPR peak observed in our study further supports the conclusion that the synthesized AgNPs predominantly fall within the 10–50 nm size range, consistent with the results obtained from transmission electron microscopy (TEM) analysis. The reduction of silver ions (Ag^+^) to elemental silver (Ag^0^) upon exposure to Hibiscus flower extract resulted in a significant color change from light pink to dark brown, which was corroborated by the emergence of a characteristic surface plasmon resonance (SPR) band centered at approximately 428 nm. The evolution of the surface plasmon resonance (SPR) band was systematically monitored over time by acquiring spectra at various time intervals throughout the reaction with an aqueous silver nitrate (AgNO_3_) solution. The hibiscus flower extract has been hypothesized to function as a capping agent, providing steric hindrance and electrostatic repulsion, which consequently inhibit the aggregation of nanoparticles and facilitate the development of a more uniform size distribution. Enhanced stabilization is evidenced by the observed color transition of the diluted AgNPs solution from light to dark brown, which occurs as the concentration of the plant extract increases. This phenomenon reflects alterations in optical properties, which are consistent with the observed spectral shifts. The plant extract serves a dual function, acting as a reducing agent that promotes the formation of Ag^0^, while concurrently functioning as a stabilizing agent that regulates growth and inhibits the aggregation of the synthesized AgNPs.

### 2.3. Morphological Characterization of HRS-AgNPs via Transmission Electron Microscopy (TEM)

Transmission electron microscopy (TEM) analysis revealed a mixture of spherical and oval morphologies, which is consistent with the characteristics of green-synthesized silver nanoparticles (AgNPs) reported in the literature [38,39]. The nanoparticles exhibited a broad size range (10–50 nm) and polydispersity, which is not uncommon for biogenic synthesis methods. This variability arises because plant-derived constituents, which act as both reducing and stabilizing agents, often vary in concentration and reactivity. Plant extracts typically contain a complex mixture of flavonoids, proteins, phenols, and other phytochemicals, which can lead to differences in nucleation and growth rates, resulting in polydispersity.

### 2.4. Dynamic Light Scattering and Zeta Potential

**Figure 2:**
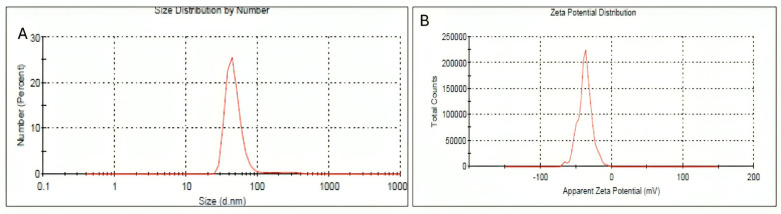
Characterization of AgNPs synthesized using *Hibiscus rosa-sinensis* extract. (**A**) Size distribution of AgNPs by number, determined by dynamic light scattering (DLS). The x-axis represents the hydrodynamic diameter of the nanoparticles in nanometers (nm), and the y-axis represents the percentage of particles at each size. (**B**) Zeta potential distribution of AgNPs. The x-axis represents the apparent zeta potential in millivolts (mV), and the y-axis represents the total number of particles at each zeta potential value.

The hydrodynamic size distribution of lyophilized HRS-AgNPs was subsequently characterized using Dynamic Light Scattering (DLS). Figure 2A illustrates the DLS data for HRS-AgNPs synthesized with 1 mL of the extract, revealing an average hydrodynamic diameter of 48 nm. Notably, the hydrodynamic diameter obtained through DLS is generally larger than the physical diameter measured by transmission electron microscopy (TEM). This discrepancy can be attributed to multiple factors, such as the influence of Brownian motion, the presence of a hydration layer surrounding the nanoparticles in solution, and the tendency of DLS to overestimate the dimensions of larger particles within the polydisperse samples. The primary advantage of DLS is its capacity to concurrently analyze a substantial population of nanoparticles [40]. The DLS data revealed a Z-average diameter of 48.20 ± 12.2% nm, with a standard deviation of 1.448 nm and a polydispersity index (PdI) of 0.420. The stability of the synthesized HRS-AgNPs was evaluated using zeta potential measurements. The measured zeta potential of approximately −22 mV (Figure 2B) indicates a moderate level of colloidal stability, which can be attributed to the electrostatic repulsion between the negatively charged nanoparticles. The observed negative charge was likely due to the adsorption of anionic biomolecules on the surfaces of the nanoparticles in the flower extract.

Despite the observed size variation, DLS measurements indicated that the HRS-AgNPs remained relatively stable, with a zeta potential of approximately –22 mV. This negative zeta potential provides electrostatic repulsion, which helps minimize particle aggregation. To assess storage stability, the AgNPs were stored at 4 °C under dark conditions for four weeks. During this period, no significant color change or precipitation was observed. Additionally, UV–Vis spectroscopy scans conducted at weekly intervals showed no notable shift in the surface plasmon resonance (SPR) peak position, further confirming the colloidal stability of the nanoparticles under these storage conditions. These findings demonstrate that green-synthesized HRS-AgNPs can maintain stability for extended periods when stored appropriately.

### 2.5. FTIR Analysis

**Figure 3:**
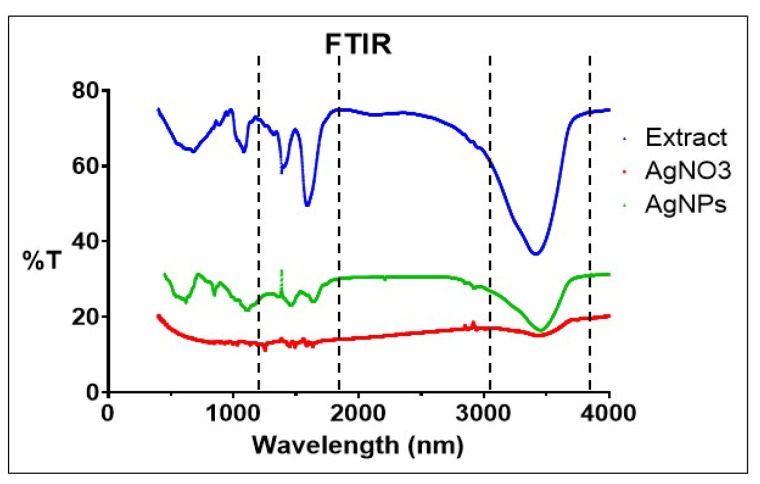
FTIR spectra of silver nanoparticles (AgNPs) synthesized using *Hibiscus rosa-sinensis* extract.

The characterization of HRS-AgNPs and the identification of functional groups involved in silver ion (Ag^+^) reduction and nanoparticle stabilization were accomplished through FTIR spectroscopy. Figure 3 illustrates the FTIR spectra of the Hibiscus flower extract (Curve A), aqueous AgNO_3_ solution (Curve B), and synthesized AgNPs (Curve C) produced by the reduction of silver nitrate via the flower extract. Distinct absorption bands in the ranges of 3,000–3,500 cm^−1^ and 1500–1680 cm^−1^ were observed in the FTIR spectrum of Hibiscus (Curve A), indicating the presence of hydroxyl (O-H) and amine (N-H) functional groups, respectively [29,30]. The stretching vibrations of O-H groups in alcoholic and phenolic compounds within the extract were attributed to the broad absorption band in the 3,000 to 3,500 cm^−1^ range. The spectrum of synthesized AgNPs (Curve C) revealed significant peaks at 3228, 3223, 3115, 3154, 3206.90, 3450, and 3486 cm^−1^, further corroborating the presence of phenolic compounds. A characteristic band in the spectral region of 1680 to 1500 cm^−1^ was attributed to the amide I band of proteins and the stretching vibrations associated with protein secondary structure [33]. The HRS-AgNPs spectrum (Curve C) revealed prominent peaks at 1632, 1640, 1513, and 1554 cm^−1^, indicative of N-C=O stretching vibrations in amide bonds of proteins and nitro compounds [33,39]. The FTIR spectrum of AgNPs displayed peaks at 1159 and 1111.02 cm^−1^, exhibiting a shift relative to the control spectrum (extract). This shift is attributable to vibrational modes of the carbonyl (C=O) functional group, suggesting its involvement in AgNP interactions. Absorption bands in the 820–550 cm^−1^ range were detected, signifying C-H deformation vibrations characteristic of synthesized silver nanoparticles (HRS-AgNPs). Spectral data analysis of the extract indicates the probable presence of flavonoids, polyphenols, and other phytochemicals, which likely contribute to AgNO_3_ reduction and AgNP synthesis. The extract spectrum exhibited absorption peaks at 3746 and 3410 cm^−1^, potentially arising from hydrogen-bonded hydroxyl (O-H) groups [41]. A peak at 2943 cm^−1^ denotes C-H stretching vibrations typical of aromatic compounds. Peaks at approximately 1753, 1622, and 1407 cm^−1^ correspond to characteristic stretching vibrations of C-H, N-H, C-C, and C-O bonds. Absorption bands at 1264 and 1077 cm^−1^ suggest the presence of C-C(=O)–O and C-O stretching vibrations associated with carboxylic acids, esters, and ethers [27,28,39]. The occurrence of similar peaks in both AgNPs and hibiscus extract spectra indicates substantial interaction between AgNPs and oxygen atoms from hydroxyl groups in compounds with high reducing sugar content within the extract. Additionally, the emergence of a new peak at 1729 cm^−1^ in the AgNP spectrum implies the formation of a novel functional group featuring a carbonyl (C=O) bond, potentially associated with aldehydes, ketones, or carboxylic acids.

### 2.6. Antibacterial Activity

**Figure 4:**
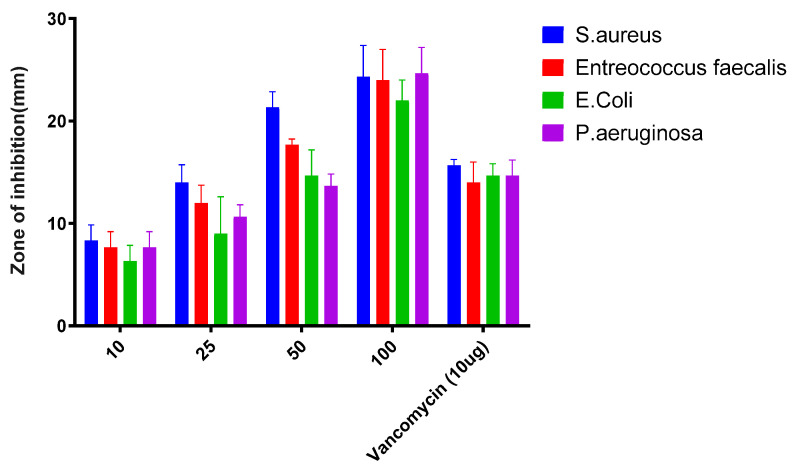
Antibacterial activity of AgNPs synthesized using *Hibiscus rosa-sinensis* extract. The figure presents the antibacterial activity of AgNPs against different gram positive and gram-negative bacterial strains: *S. aureus*, *Enterococcus faecalis*, *E. coli*, and *P. aeruginosa* at different concentrations of HRS-AgNPs used in the assay (10, 25, 50, 100 µg/mL) and a comparison to the standard antibiotic Vancomycin (10 µg). The experiments were performed in triplicate.

The antibacterial efficacy of the synthesized HRS-AgNPs was evaluated against clinically relevant bacterial pathogens. The results, as presented in Figure 4, demonstrated that HRS-AgNPs exhibited a significant inhibitory effect against all tested strains, with statistically significant (*p* < 0.05) differences observed in the diameters of the zones of inhibition. Notably, HRS-AgNPs demonstrated a more pronounced antagonistic effect against gram-positive bacterial strains, with inhibition zones ranging from 22 ± 1.5 to 24 ± 4.4 mm in *S. aureus* and *E. faecalis* at a dose of 100 µg/mL. Conversely, the activity against gram-negative strains was comparatively lower, with inhibition zones measuring 16 ± 3.05 to 15 ± 2.5 mm against *E. coli* and *P. aeruginosa* at a dose of 100 µg/mL. These findings are consistent with previous studies conducted by El-Batal et al. [39,42,43], which indicated that the antimicrobial activity of in situ synthesized HRS-AgNPs was also assessed following 96-well plate dilution methods. Vancomycin, a standard antibiotic, was used as a control for comparison. The Minimum Inhibitory Concentration (MIC) values of the synthesized AgNPs were found to vary significantly depending on the microorganism tested. The MIC for *S. aureus* and *E. faecalis* was found to be 32 and 128 µg/mL, and for the gram-negative *E. coli* and *P. aeruginosa* it was around 64 µg/mL each. These results highlight the differential sensitivity of the tested strains to the nanoparticles, underscoring the potential of AgNPs as an effective antimicrobial agent.

Gram-negative bacteria exhibit reduced susceptibility to HRS-AgNPs compared to Gram-positive bacteria. The antibacterial mechanism of silver nanoparticles is multifaceted, involving the perforation of bacterial cell walls, disruption of cell membrane integrity through the release of Ag^2+^ ions, and induction of DNA damage, ultimately leading to the inhibition of bacterial growth and cell death. Furthermore, the intracellular accumulation of Ag^2+^ ions has been shown to induce the formation of reactive oxygen species (ROS), which can compromise bacterial viability by targeting and degrading lipid membranes as well as inhibiting protein synthesis. This mechanistic paradigm was corroborated by Rauf et al. [24,25,44], who used scanning electron microscopy (SEM) and transmission electron microscopy (TEM) to demonstrate that AgNPs initially disrupt the structural integrity of bacterial cell membranes. This disruption facilitates their penetration into the cytoplasm, where they interact with multiple subcellular components, ultimately resulting in bacterial cell death. The clinical significance of biofilms is attributed to their capacity to impede the efficacy of conventional antibiotics, a phenomenon primarily due to the protective exopolysaccharide (EPS) matrix that they produce. The continuous administration of antibiotics for the management of biofilm-associated infections has facilitated the development of antibiotic resistance among various bacterial pathogens, consequently prolonging treatment duration and increasing the probability of treatment failure. Consequently, clinicians have been increasingly reliant on combination antibiotic therapy. However, persistent utilization of this approach may exacerbate the critical issue of antibiotic resistance in clinically significant pathogens. Nanomaterials, particularly metal nanoparticles, have garnered substantial interest because of their effective inhibitory effects on bacterial growth and biofilm formation, a phenomenon that can be attributed to their elevated surface area-to-volume ratio [37,45].

**Figure 5:**
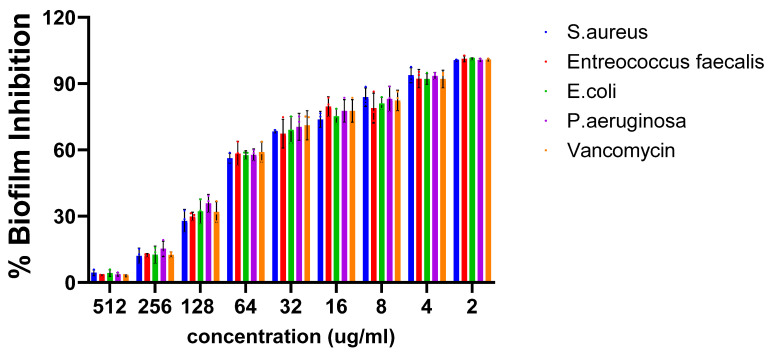
Effect of HRS-AgNPs on biofilm formation by different bacterial strains. This figure illustrates the impact of HRS-AgNPs synthesized using *Hibiscus rosa-sinensis* extract on the biofilm formation of four bacterial strains: *E. coli*, *P.aeruginosa*, *S. aureus*, and *E. faecalis*.

The biofilm inhibitory efficacy of HRS-AgNPs was evaluated against both Gram-positive and Gram-negative bacterial pathogens, as illustrated in Figure 5. The results revealed a significant dose-dependent inhibition of biofilm formation across all examined pathogens. Notably, Gram-positive strains exhibited superior biofilm inhibition, with *S. aureus* and *E. faecalis* demonstrating inhibition rates of 92% and 90%, respectively. In contrast, Gram-negative bacteria showed lower inhibition rates, with *E. coli* at 84% and *P. aeruginosa* at 82% at a concentration of 256 μg/mL. This differential susceptibility may be attributed to the thicker peptidoglycan layer in Gram-positive bacteria, potentially facilitating enhanced nanoparticle interaction and penetration. Conversely, the outer membrane of Gram-negative bacteria, primarily composed of lipopolysaccharide (LPS), serves as an additional barrier. Nevertheless, the observed significant inhibition of biofilm formation in Gram-negative bacteria by AgNPs suggests their ability to overcome this barrier or employ alternative mechanisms for biofilm disruption.

The interaction between nanoparticles and biofilms is proposed to occur in three stages: initial surface interaction, matrix adherence, and biofilm penetration [7,22,37]. Infiltrated nanoparticles are hypothesized to interfere with quorum-sensing signaling molecules, such as N-acyl homoserine lactones (AHLs) in Gram-negative bacteria and oligopeptides in Gram-positive bacteria [38,39,40], potentially disrupting coordinated biofilm formation regulation. The extent of nanoparticle infiltration is largely influenced by electrostatic interactions and the physicochemical properties of both the EPS matrix and the nanoparticles. The observed biofilm inhibitory activity of AgNPs suggests a possible destabilization of the biofilm matrix, attributable to their inherent antibacterial properties. These findings align with previous studies demonstrating the significant antibacterial and biofilm inhibitory capabilities of silver nanoparticles against *S. aureus*, *P. aeruginosa*, *E. coli*, and *B. subtilis* [42,43,44]. Furthermore, AgNPs have been reported to exhibit safety for human cellular structures while maintaining their antibacterial and biofilm inhibitory properties. The substantial antibiofilm properties of HRS-AgNPs can be ascribed to the induction of elevated reactive oxygen species (ROS) production, coupled with DNA and cell membrane damage, ultimately leading to cellular death. Our research findings indicate that the application of silver nanoparticles prior to biofilm establishment can significantly reduce the planktonic cell population, thereby decreasing the susceptibility of abiotic surfaces to bacterial adhesion.

### 2.7. Cytotoxic Potential

**Figure 6:**
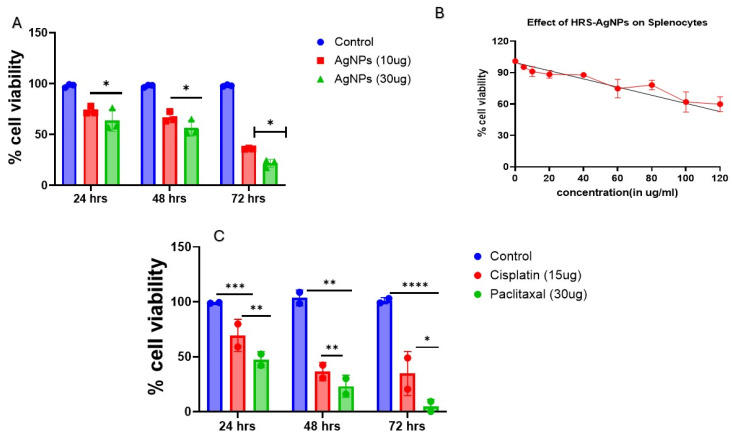
Evaluation of HRS-AgNPs cytotoxicity. The figure depicts the outcomes of a cytotoxicity assay examining the impact of silver nanoparticles synthesized from *Hibiscus rosa-sinensis* extract on MDMB-231 cells and splenocytes. (**A**) MDMB-231 cell viability assessed at 24-, 48-, and 72-h post-exposure to 15 and 30 μg/mL of AgNPs. (**B**) Concentration-dependent effects of AgNPs on splenocyte viability. Experiments were replicated thrice, with error bars representing standard error of the mean (SEM). (**C**) MDMB-231 cell viability assessed at 24-, 48-, and 72-h post-exposure to 15 and 30 μg/mL of cisplatin and paclitaxal. Statistical significance: * *p* < 0.05, ** *p* < 0.01 relative, *** *p* < 0.001, **** *p* < 0.0001 to control conditions.

Next, we evaluated the cytotoxic effect of HRS-AgNPs on the MDMB-231 cell line. Utilizing an MTT-based cell viability assay over a 24 to 72 h exposure period, as depicted in Figure 6A, the study revealed a time- and dose-dependent decrease in cell viability. Notably, at 30 μg/mL concentration, AgNPs induced a 51% reduction in MDMB-231 cell viability after 48 h, compared to a 24% decrease at 72 h. To assess the therapeutic selectivity of HRS-AgNPs, their effects were evaluated in non-tumorigenic splenocytes. As illustrated in Figure 6B, HRS-AgNPs demonstrated substantially higher cytotoxicity toward MDMB-231 cells relative to splenocytes. Remarkably, the splenocytes exhibited no significant reduction in viability throughout the experimental period. Following 24-h exposure to 30 μg/mL AgNPs, splenocytes maintained an approximately 83% survival rate, whereas MDMB-231 cells displayed a markedly lower 69% survival rate. These findings suggest that HRS-AgNPs exhibit a degree of selective cytotoxicity, predominantly affecting the MDMB-231 cancer cell line, while demonstrating minimal toxicity toward non-tumorigenic splenocytes. This study emphasizes the importance of evaluating nanoparticle-based therapies in oncology, particularly focusing on the capacity of AgNPs to selectively target malignant cells while minimizing the adverse effects on healthy non-transformed cells. This selectivity underscores the potential of AgNPs as promising candidates for developing advanced cancer therapeutics with enhanced therapeutic indices.

**Figure 7:**
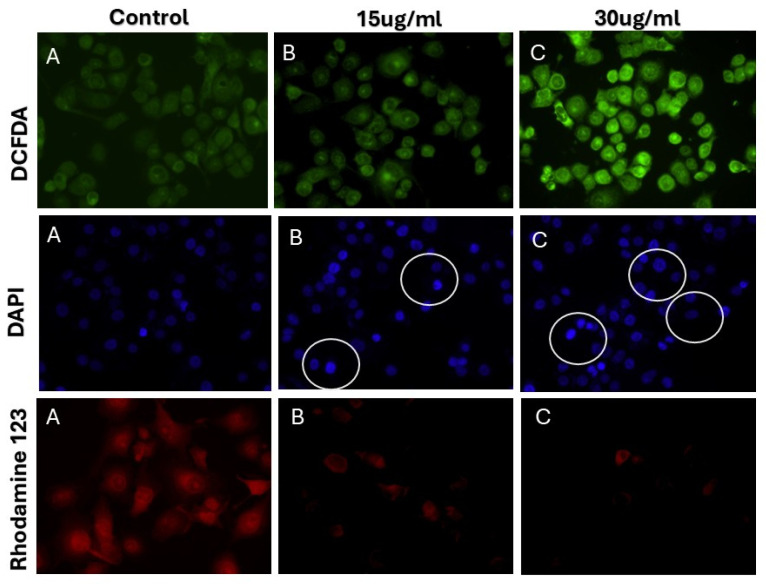
Fluorescence Microscopy Images of MDMB-231 cells treated with HRS-AgNPs: The fluorescence microscopy images of MDMB-231 cells following treatment with varying concentrations of *Hibiscus rosa-sinensis* extract-synthesized AgNPs. Each row showcases a different fluorescent stain: DCFDA (green) for detecting ROS production, indicative of oxidative stress; DAPI (blue) for staining cell nuclei to visualize cell count and morphology; and Rhodamine 123 (red) for assessing mitochondrial function by its accumulation in active mitochondria. The columns represent different treatment conditions: Control (**A**) with untreated cells, 15 µg/mL HRS-AgNPs (**B**), and 30 µg/mL HRS-AgNPs (**C**). Observations from the images reveal a potential increase in green fluorescence (ROS production) with higher AgNPs concentrations, suggesting a dose-dependent induction of oxidative stress. The white circles in the DAPI-stained images highlight possible alterations in cell numbers, nuclear morphology, or signs of apoptosis in the treated groups. Additionally, a decrease in red fluorescence (mitochondrial activity) with increasing HRS-AgNPs concentrations may indicate mitochondrial dysfunction. These findings correlate with the reduced cell viability observed in Figure 6, suggesting that AgNPs induce oxidative stress and mitochondrial dysfunction in splenocytes, ultimately contributing to cell death.

The assessment of intracellular ROS was conducted utilizing the 2′,7′-dichlorodihydrofluorescein diacetate (DCFH-DA) assay, a well-established method for the indirect quantification of cellular hydrogen peroxide (H_2_O_2_) production. As illustrated in Figure 7A, MDMB-231 cells exposed to HRS-AgNPs exhibited a statistically significant enhancement in intracellular ROS generation relative to the untreated control. This elevation in ROS levels demonstrated a clear dose-dependent correlation, with higher concentrations of HRS-AgNPs eliciting a more pronounced increase compared to the control group. These findings align with our previous research and studies by Akhtar et al. and Li et al., which revealed that AgNPs induce cytotoxic and genotoxic effects in primary Syrian hamster embryo (SHE) cells, primarily through the disruption of cell cycle progression and inhibition of cellular replication. Such observations highlight the pivotal role of ROS in mediating the cytotoxic effects of silver nanoparticles (AgNPs) and their involvement in triggering apoptotic signaling cascades [46].

The potential of the HRS-AgNPs to induce apoptosis was investigated using the cell-permeable nuclear stain Hoechst 33342. The dye has a binding affinity for adenine-thymine (A-T) rich regions within DNA, facilitating the visualization of nuclear morphology and enabling the identification of apoptotic characteristics. After a 24-h exposure to HRS-AgNPs (15 and 30 μg/mL), MDMB-231 cells were subjected to staining with Hoechst 33342 and subsequently analyzed via fluorescence microscopy. The untreated control cells displayed uniformly stained spherical nuclei, maintaining a coherent nuclear architecture (Figure 7B). In contrast, cells subjected to treatment with HRS-AgNPs exhibited notable morphological alterations indicative of apoptosis. The treated cells showed significant chromatin condensation, nuclear fragmentation, and the emergence of apoptotic bodies, which are membrane-bound vesicles containing fragmented nuclear material.

Mitochondrial dysfunction is a recognized precursor to the onset of the apoptotic cascade. This study quantitatively assessed the activity of mitochondrial membrane potential (ΔΨm) in MDMB-231 cells exposed to different HRS-AgNPs. The mitochondrial membrane potential (ΔΨm) was assessed using fluorescence microscopy (FM) after staining with rhodamine 123. A decrease in rhodamine 123 fluorescence intensity indicates a reduction in ΔΨm, representing an initial event in the apoptotic process. As demonstrated in Figure 7C, cells subjected to HRS-AgNPs treatment exhibited a significant reduction in ΔΨm compared to the untreated control group. The cohort that was administered 30 μg/mL of HRS-AgNPs demonstrated the most substantial decrease in ΔΨm. The observed decline in ΔΨm is hypothesized to be associated with the induction of mitochondrial permeability transition pore (MPTP) opening, which is a pivotal event that facilitates the progression of cellular apoptosis. The opening of the mitochondrial permeability transition pore (MPTP) enables the non-selective influx of solutes with molecular weights reaching 1500 Da into the mitochondrial matrix, resulting in mitochondrial depolarization and uncoupling of oxidative phosphorylation processes. This bioenergetic compromise leads to a significant reduction in adenosine triphosphate (ATP) production, which is further exacerbated by the continuous process of ATP hydrolysis [47,48]. Moreover, prior studies have demonstrated a correlation between the opening of the MPTP, a reduction in ΔΨm, increased intracellular calcium ion (Ca^2+^) levels, and subsequent activation of downstream apoptotic signaling pathways. The results of the current study align with these established studies, thereby corroborating the involvement of HRS-AgNPs in the induction of apoptosis through the disruption of mitochondrial homeostasis. These findings elucidate the potential of HRS-AgNPs as efficacious colloidal agents in anticancer therapy and present a promising direction for the advancement of innovative therapeutic strategies targeting the mitochondrial apoptotic pathway.

**Figure 8:**
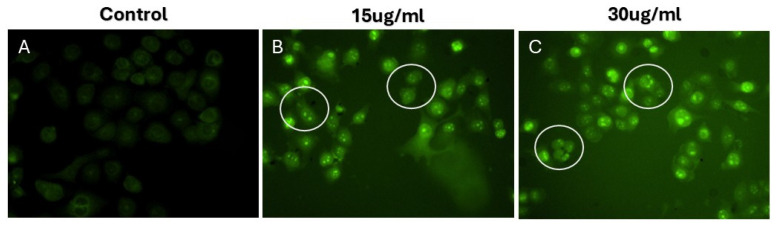
Caspase-3 activity in MDMB-231 cells treated with HRS-AgNPs: The figure illustrates the activity of caspase-3, a crucial executioner caspase in apoptosis (programmed cell death), within cells following treatment with various doses of HRS-AgNPs. Each column represents a different treatment condition: Control (**A**) without AgNPs, 15 µg/mL AgNPs (**B**), and 30 µg/mL AgNPs (**C**). Observations from the figure indicate a clear increase in caspase-3 activity in cells treated with HRS-AgNPs, particularly at the higher concentration (30 µg/mL), as evidenced by the stronger green fluorescence. The white circles highlight areas with notably increased caspase-3 activity in the AgNP-treated groups. This observation corroborates the findings in Figure 6 and Figure 7, which demonstrated decreased cell viability, increased ROS production, and mitochondrial dysfunction in cells exposed to AgNPs. The activation of caspase-3 confirms that apoptosis is a key mechanism of AgNP-induced cytotoxicity.

Apoptosis, a genetically controlled process of programmed cell death, is characterized by the methodical breakdown of intracellular components while concurrently mitigating inflammation and safeguarding neighboring cells [49,50]. The caspase family, comprising cysteine aspartate-specific proteases, plays a crucial role in orchestrating inflammatory responses and the apoptotic sequence [51,52]. These caspases are categorized into two main functional groups: initiator caspases (including caspases 2, 8, 9, and 10) and effector caspases (encompassing caspases 3, 6, and 7) [51,52,53]. The apoptotic cascade is typically set in motion by the interplay between caspase-3 and the initiator caspases-8 and -9. This interaction marks a decisive juncture, after which the apoptotic process becomes irreversible. In order to elucidate the mechanisms by which HRS-AgNPs induce apoptosis, the activity levels of caspase-3 were assessed in MDMB-231 cells exposed to HRS-AgNPs at concentrations of 15 and 30 µg/mL. Caspase-3, a crucial mediator of programmed cell death in cancer cells, is activated by various apoptotic stimuli. As illustrated in Figure 8, caspase-3 activity exhibited a three-fold increase in cells treated with 30 µg/mL HRS-AgNPs for 48 h compared to untreated controls. These findings are consistent with previous research conducted by Kikuchi et al. and Arland et al. [54,55]. Additionally, the observed morphological alterations in both the cell membrane and nucleus provide supporting evidence for AgNP-induced apoptosis in cancer cells [37,56]. During the apoptotic process, initiator caspases, including caspase-3, are synthesized as inactive zymogens in the cytoplasm, poised to initiate programmed cell death upon activation [57,58]. Exposure to AgNPs triggers the activation of specific caspases while simultaneously promoting the generation of reactive oxygen species (ROS), as depicted in Figure 8A. This cascade of events leads to DNA damage, endoplasmic reticulum (ER) stress, and protein misfolding, ultimately resulting in apoptosis. Activated caspase-3 cleaves and activates caspase-activated DNase (CAD), causing fragmentation of genomic DNA. Endonucleolytic DNA fragmentation is widely acknowledged as a characteristic biochemical marker of apoptosis, typically occurring during the early stages of this process [42,43,44]. Comparable observations were reported by Arora et al. in their investigation of cellular responses to silver nanoparticle exposure [59].

## 3. Materials and Methods

### 3.1. Hibiscus Rosa Sinensis Flower Extract Preparation and Synthesis of Silver Nanoparticles

The preparation of an aqueous extract from *Hibiscus rosa-sinensis* (HRS) flowers for experimental use followed a standardized protocol adapted from previous research [28]. Fresh HRS flowers underwent thorough washing to remove potential contaminants, followed by air-drying on a muslin sheet for 72 h. A precise quantity of cleaned flowers (30 g) was finely minced and boiled in 100 mL of sterile distilled water to facilitate extraction. The resulting decoction, termed the HRS flower extract, was filtered through Whatman filter (Fischer Scientific, Pittsburgh, PA, USA) paper to eliminate particulates. The supernatant was then isolated for further experimentation.

The ratio of plant extract to AgNO_3_ is critical for achieving reproducible nanoparticle size, shape, and yield, and preliminary experiments with varying volumes of *H. rosa sinensis* flower extract (0.5 mL, 1 mL, and 2 mL) against a fixed concentration of AgNO_3_ (1 mM) revealed that a 1:3 ratio (extract to AgNO_3_ solution) yielded the most consistent results, producing nanoparticles with a narrower size distribution and a stable surface plasmon resonance (SPR) peak, as ratios below 1:3 tended to produce incomplete reduction or unstable suspensions, while higher ratios occasionally led to excessive capping and broader size distributions. To ensure replicability, the reaction mixture was adjusted to pH 7.0 ± 0.2 before the addition of the plant extract, as this near-neutral pH offered a balance between efficient reduction and avoiding excessive aggregation, and all reactions were carried out at 25 ± 2 °C (room temperature), as higher temperatures (>40 °C) led to rapid particle growth and broader polydispersity. The reaction mixture was stirred at 300 rpm for the first 10 min to facilitate uniform mixing of the extract and silver nitrate, then allowed to stand without agitation, as continuous stirring for the entire reaction sometimes introduced unwanted secondary nucleation events, leading to less uniform particles, and the total reaction time was 24 h, with samples intermittently evaluated (at 1, 3, 6, and 24 h) by UV–Vis spectroscopy to track the development and stabilization of the SPR peak. By systematically optimizing and documenting these parameters, we achieved reproducible AgNPs synthesis using *H. rosa sinensis* extract, providing an approach that can be adopted or further refined by other researchers to ensure consistent results. Visual changes in the reaction mixture were monitored at 30-min intervals. The formation of AgNPs, indicated by the reduction of silver ions (Ag^+^) to zerovalent silver (Ag^0^), was qualitatively evaluated through observation of a progressive color change in the solution.

### 3.2. Absorption Spectra of Silver Nanoparticles (AgNPs)

Silver nanoparticles (AgNPs) were synthesized via a bioreduction method utilizing *Hibiscus rosa-sinensis* flower extract and characterized by ultraviolet-visible (UV–Vis) absorption spectroscopy, focusing on their formation and optical properties. The spectra were obtained using a Jasco V-750 double-beam spectrophotometer (Easton, MD, USA) operating at a spectral resolution of 1 nm. The analysis was conducted within the wavelength range appropriate for the observation of the Surface Plasmon Resonance (SPR) band characteristic of AgNPs, which typically spans from 300 to 800 nm. This methodology enables the evaluation of nanoparticle formation, dimensions, and stability because these factors significantly influence the position and morphology of the SPR band [60,61,62].

### 3.3. Fourier-Transform Infrared Spectroscopy (FT-IR) Measurements

FTIR spectroscopy was employed to characterize the functional groups present in the extract of HRS flowers and biogenically synthesized AgNPs. FTIR spectra were obtained using a Perkin-Elmer Spectrum One FTIR spectrophotometer (Shelton, CT, USA), equipped with an attenuated total reflectance (ATR) accessory and operated in the diffuse reflectance mode. The spectra were acquired in the wavenumber range of 4000 cm^−1^ to 900 cm^−1^, utilizing a spectral resolution of 4 cm^−1^. To ensure data reproducibility and minimize noise, each sample underwent three scanning iterations, with each spectrum representing the average of 128 co-added scans. The samples subjected to analysis comprised the HRS flower extract in isolation, an aqueous solution of silver nitrate (AgNO_3_, used as a control), and the synthesized AgNPs produced from the interaction between the HRS flower extract and AgNO_3_ [60,63,64].

### 3.4. Transmission Electron Microscopy (TEM) Measurements

TEM was employed to elucidate the morphological features and dimensional attributes of AgNPs synthesized through a biogenic process. The synthesis utilized HRS flower extract, which served dual roles as a reducing and stabilizing agent. In preparation for TEM analysis, a minute quantity of the AgNPs reaction mixture was deposited onto a gold-coated copper grid. The sample was subsequently allowed to desiccate under ambient conditions, yielding a thin nanoparticle film on the grid surface [32]. TEM imaging was performed using a JEOL JEM-2100 Plus instrument (Tokyo, Japan), operated at a 200 kV accelerating voltage.

### 3.5. In Vitro Antibacterial Activity

The agar well diffusion technique was employed to assess antibacterial activity. Four bacterial strains were examined: *Staphylococcus aureus* and *Enterococcus faecalis* (gram-positive), along with *Escherichia coli* and *Pseudomonas aeruginosa* (gram-negative). These organisms were primarily obtained from the Hail hospital and the Biology department. Each bacterial strain (*Staphylococcus aureus*, *Enterococcus faecalis*, *Escherichia coli*, and *Pseudomonas aeruginosa*) was initially cultured in nutrient broth at 37 °C for 18–24 h. The cultures were then standardized to a density of 1.5 × 10^8^ CFU/mL (equivalent to a 0.5 McFarland standard) by adjusting turbidity using sterile saline. Subsequently, 100 µL of the standardized suspension was uniformly spread onto nutrient agar plates prior to well diffusion. This inoculum size ensured reproducible and consistent growth conditions across all tested strains to evaluate their susceptibility. HRS-AgNPs were applied at varying concentrations. The plates were then incubated at 37 °C for a 24-h period. Subsequently, the degree of antibacterial efficacy was determined by measuring the zones of inhibition around the wells [65,66,67]. Minimum Inhibitory Concentration (MIC) is defined as the lowest concentration of an antimicrobial agent that inhibits the visible growth of microorganisms after a specified incubation period. MIC is a critical parameter used in diagnostic laboratories to evaluate the susceptibility of microorganisms to specific antimicrobial agents. In this study, the MIC values of in situ synthesized AgNPs were determined using the microdilution method against *Staphylococcus aureus* and *Enterococcus faecalis* (gram-positive), along with *Escherichia coli* and *Pseudomonas aeruginosa* (gram-negative). The MIC values were determined using a viability test conducted in 96-well microdilution plates, following established protocols.

### 3.6. Evaluation of the Antibiofilm Activity of Silver Nanoparticles Through XTT Reduction Assay

The efficacy of the synthesized HRS-AgNPs in combating biofilms was examined using the 2,3-bis-(2-methoxy-4-nitro-5-sulfophenyl)-2H-tetrazolium-5-carboxanilide (XTT) reduction assay. This colorimetric technique is frequently used to assess the metabolic activity and viability of biofilms. The experimental procedure involved initial biofilm formation in 96-well microtiter plates. Following the primary biofilm development phase, thorough washing with sterile phosphate-buffered saline PBS (pH 7.4) was performed to remove planktonic and loosely adherent cells. The established biofilms were subsequently exposed to varying concentrations of HRS-AgNPs for 48 h, enabling the assessment of the capacity of AgNPs to inhibit mature biofilms. After treatment, XTT solution, which undergoes reduction by metabolically active cells to produce a colored formazan product, was introduced into each well. After an appropriate incubation period to allow for color development, the optical density (OD) of each well was spectrophotometrically measured at 490 nm. The observed decrease in OD relative to untreated controls serves as an indicator of the antibiofilm activity of HRS-AgNPs [68].

### 3.7. Assessment of Cytotoxicity Utilizing the 3-(4,5-Dimethylthiazol-2-yl)-2,5-Diphenyltetrazolium Bromide (MTT) Assay

The evaluation of HRS-AgNPs cytotoxicity employed the 3-(4,5-dimethylthiazol-2-yl)-2,5-diphenyltetrazolium bromide (MTT) reduction assay, a colorimetric technique that assesses mitochondrial dehydrogenase activity as an indicator of cell viability. This protocol was adapted from the method outlined by Rauf et al. [62] with slight modifications. MDMB-231 cells were cultured in 96-well plates and allowed to attach overnight. The cells were then treated with varying concentrations of HRS-AgNPs for different time periods. After treatment, MTT solution was added to each well and incubated, allowing metabolically active cells to convert the yellow tetrazolium salt (MTT) into a purple formazan product. Following solubilization of the formazan crystals, the absorbance was measured spectrophotometrically at 490 nm [69,70].

### 3.8. Quantification of Intracellular Reactive Oxygen Species (ROS)

Intracellular production of reactive oxygen species (ROS) induced by HRS-AgNPs was assessed using fluorescence microscopy (FM) in conjunction with the oxidation-sensitive fluorogenic probe DCFH-DA. This assay is based on the principle that cell-permeable DCFH-DA undergoes intracellular deacetylation by esterases to form non-fluorescent DCFH, which is subsequently oxidized by ROS, primarily hydrogen peroxide, to highly fluorescent 2′,7′-dichlorofluorescein (DCF). The intensity of the emitted fluorescence was directly proportional to intracellular ROS levels, and the experimental procedure was adapted from the established protocol described by Eruslanov et al. [71] with minor modifications. Briefly, cells were seeded in 6 well plates and allowed to adhere overnight. Subsequently, cells were exposed to varying concentrations of HRS-AgNPs for 24 h. Following treatment, the cells were incubated with DCFH-DA for a specific duration to facilitate cellular uptake and deacetylation. After incubation, the cells were washed to remove excess probe and the resultant fluorescence was visualized under a fluorescence microscope.

### 3.9. Evaluation of Morphological Changes Utilizing 4′,6-Diamidino-2-Phenylindole (DAPI) Staining

The potential of HRS-AgNPs to induce apoptosis-related morphological changes was investigated in MDMB-231 cells. These cells were seeded in 6-well plates at a concentration of 1 × 10^5^ cells per well. After allowing time for adherence, cells were treated with HRS-AgNPs at two different concentrations (15 and 30 μg/mL) for a 24-h period under standard incubation conditions (37 °C, 5% CO_2_). To preserve cellular structure, the cells were fixed using 4% paraformaldehyde for 15 min at room temperature. Following fixation, the cells were washed with phosphate-buffered saline PBS (pH 7.4) to eliminate any remaining fixative. Nuclear morphology was visualized by staining the cells with 4′,6-diamidino-2-phenylindole (DAPI), a DNA-binding fluorescent dye, at 1 μg/mL in PBS for 10 min. Excess dye was removed by two subsequent PBS washes. A fluorescence microscope with suitable filters for DAPI fluorescence was used to assess morphological changes and apoptotic features, such as chromatin condensation and nuclear fragmentation. This approach, adapted from methods described by Ferro et al., enabled the systematic examination and documentation of observable nuclear morphological alterations in cells exposed to various concentrations of HRS-AgNPs [41].

### 3.10. The Evaluation of Mitochondrial Membrane Potential (ΔΨm)

To assess the impact of biogenically synthesized HRS-AgNPs on mitochondrial membrane potential (ΔΨm), which serves as a vital indicator of mitochondrial functionality and cellular health, the cationic fluorophore Rhodamine 123 was utilized. MDMB-231 cells were initially seeded into a 6 well plate and, after achieving adherence, were subjected to different concentrations of AgNPs for 24 h. Following this, the cells were fixed using 4% paraformaldehyde for 30 min at 25 °C to maintain cellular morphology. Subsequent to fixation, the cells underwent a washing procedure utilizing phosphate-buffered saline to eliminate any residual fixative present. To evaluate the mitochondrial membrane potential (ΔΨm), cells were incubated with rhodamine 123 at a concentration of 5 μg/mL in phosphate-buffered saline (PBS; BD Biosciences, Franklin Lakes, NJ, USA) for 30 min at 37 °C in the dark.

Rhodamine 123 is a cationic dye that permeates cell membranes and accumulates within the mitochondrial matrix of healthy cells, a process facilitated by a negative electrochemical gradient that exists across the inner mitochondrial membrane. A reduction in rhodamine 123 fluorescence intensity serves as an indicator of diminished ΔΨm, which signifies mitochondrial depolarization and possible dysfunction. After incubation with the dye, the cells were washed twice with phosphate-buffered saline (PBS) to eliminate any unbound dye residues. The fluorescence intensity of the stained cells was subsequently visualized and qualitatively assessed using a fluorescence microscope (excitation/emission wavelength: 488/520 nm) at a magnification of 10× [72,73].

### 3.11. The Evaluation of Cleaved Caspase 3 Through Fluorescence Microscopy

The evaluation of HRS-AgNPs’ effect on cellular apoptosis was conducted using the caspase 3 dye. MDMB-231 cells were initially plated in 6-well plates and allowed to adhere before being subjected to varying concentrations of AgNPs for a 24-h period. To preserve cellular structure, the cells were then fixed with 4% paraformaldehyde for 30 min at room temperature (25 °C). A subsequent washing step with phosphate-buffered saline was performed to remove any residual fixative. Following dye incubation, the cells underwent two additional washes with phosphate-buffered saline (PBS) to eliminate excess unbound dye. Fluorescence microscopy at 10X magnification was then employed to visualize and qualitatively assess the fluorescent intensity of the stained cells.

**Statistical Analysis:** Results were considered statistically significant at *p* < 0.05. All experiments were performed in triplicate, and data are presented as mean ± standard error mean.

## 4. Conclusions, Limitations, and Future Directions

The present investigation presents an eco-friendly and biogenic approach for the synthesis of HRS-AgNPs using aqueous extracts derived from *Hibiscus rosa sinensis*. This method offers a sustainable alternative for producing silver nanostructures, yielding AgNPs with a polydisperse size distribution ranging from 10 to 100 nm, predominantly spherical morphology, and a negative zeta potential, indicative of colloidal stability. The synthesized AgNPs exhibited remarkable antibacterial properties, demonstrating enhanced efficacy against both Gram-positive and Gram-negative bacteria. Furthermore, these nanoparticles displayed significant cytotoxicity toward MDMB-231 cells while exerting minimal effects on splenocytes, suggesting a degree of tumor selectivity. HRS-AgNPs’ antiproliferative actions are probably mediated by caspase activation, mitochondrial malfunction, and oxidative stress induction among other processes. Our results show that HRS-AgNPs greatly boost the generation of reactive oxygen species (ROS) in MDMB-231 cells, hence causing cellular damage and activating death-signalling pathways. Furthermore, the noted drop in mitochondrial membrane potential (ΔΨm) points to HRS-AgNPs causing mitochondrial malfunction, which could release pro-apoptotic proteins and activate caspases. The notable rise in caspase-3 activity even further supports the participation of apoptosis in HRS-AgNP-induced cell death. These findings collectively suggest that HRS-AgNPs exert their antiproliferative effects through a multifaceted mechanism involving oxidative stress, mitochondrial dysfunction, and caspase-dependent apoptosis

This study provides compelling evidence for the diverse biological activities of biogenically synthesized AgNPs, highlighting their potent antibacterial properties and potential as a novel therapeutic strategy for cancer treatment. The findings presented herein contribute significantly to the fields of nanomedicine and bioengineering, offering fresh insights into the application of nanotechnology in healthcare and the development of innovative therapeutic modalities. This study lays the groundwork for future investigations aimed at optimizing AgNP synthesis, elucidating the precise molecular mechanisms underlying their biological activities, and evaluating their therapeutic efficacy and safety in vivo.

### Limitations and Future Directions

While the current study demonstrates the promising antibacterial and anticancer efficacy of *Hibiscus rosa sinensis*-mediated silver nanoparticles, several limitations should be acknowledged. First, the nanoparticles exhibited a relatively broad size distribution due to the complex nature of plant-derived reducing and stabilizing agents, which may affect batch-to-batch reproducibility. Second, cytotoxic evaluations were limited to one breast cancer cell line (MDMB-231) and normal splenocytes; expanding these assessments to include additional tumor types and a broader panel of normal cells would provide a more comprehensive safety profile. Finally, in vivo studies are essential to confirm the pharmacokinetics, biodistribution, and potential off-target effects of these biogenic nanoparticles. Moving forward, future research should focus on refining synthesis methods to achieve tighter control over nanoparticle size and morphology, potentially through standardization or fractionation of the plant extract. Investigating the molecular mechanisms underlying their antibacterial and anticancer activities, such as gene expression changes, apoptotic regulators, and immune responses, will help elucidate their modes of action. Additionally, in vivo efficacy and toxicity studies in appropriate animal models will be critical for validating clinical potential. Exploring combination therapies, such as integrating these green-synthesized AgNPs with conventional antibiotics or chemotherapeutics, could also yield synergistic effects while minimizing adverse outcomes.

## Data Availability

Data is contained within the article.
